# Late adverse events in patients with pelvic cancer after oncologic treatment—intervention and treatment effect

**DOI:** 10.1007/s00520-023-07733-3

**Published:** 2023-04-13

**Authors:** Sofia Iselius, Annica Knutsen, Rasmus Mikiver, Srinivas Uppugunduri

**Affiliations:** 1 Department of Clinical and Experimental Medicine, Linköping University, Linköping, Sweden; 2 Department of Oncology, Linköping University, S-58185 Linköping, Sweden; 3 Regional Cancer Centre Southeast, Linköping, Sverige

**Keywords:** Gastrointestinal, Urologic, Sexual, Late side effects, Oncologic treatment, Pelvic cancer

## Abstract

**Purpose:**

Few studies have focused on the late adverse events after oncologic treatment in pelvic cancer patients. Here, the treatment effect/interventions were studied on late side effects as GI, sexual, and urinary symptoms in pelvic cancer patients who visited a highly specialized rehabilitation clinic in Linköping.

**Methods:**

This retrospective longitudinal cohort study included 90 patients who had at least one visit at the rehabilitation clinic for late adverse events at Linköping University hospital between 2013 to 2019. The toxicity of the adverse events was analyzed by using the common terminology criteria for adverse events (CTCAE).

**Results:**

By comparing the toxicity of symptoms between visits 1 and 2, we showed that the GI symptoms decreased with 36.6% (*P* = 0.013), the sexual symptoms with 18.3% (*P* < 0.0001), and urinary symptoms with 15.5% (*P* = 0.004). Patients who received bile salt sequestrant had a significant improvement in grade of GI symptoms as diarrhea/fecal incontinence at visit 2 compared to visit 1 where 91.3% were shown to have a treatment effect (*P* = 0.0034). The sexual symptoms (vaginal dryness/pain) significantly improved due to local estrogens between visits 1 and 2 where 58.1% had a reduction of symptoms (*P* = 0.0026).

**Conclusion:**

The late side effects as GI, sexual, and urinary symptoms was significantly reduced between visits 1 and 2 at the specialized rehabilitation center in Linköping. Bile salt sequestrants and local estrogens are effective treatments for side effects as diarrhea and vaginal dryness/pain.

## Introduction

Gynecologic cancer is the second most common cancer in the world among women, and in Sweden, around 3000 women are diagnosed each year. Today, most gynecologic cancers are associated with a high 5-year survival of around 80%. An increasing incidence in cancer, coupled with improved treatments, has resulted in a higher number of patients surviving their disease, and therefore, there is a growing need to improve the patients’ quality of life after oncologic treatment.

Treatment for pelvic cancer often includes high doses of radiotherapy directed to the pelvic area. Many of these patients develop late side effects such as chronic changes of the gastrointestinal (GI) tract, and for around 50% of these patients, the symptoms affect their daily activity and quality of life [1, 2]. In previous reports, roughly twenty symptoms from the GI tract were described and in 20–30% of these cases, two or more symptoms existed concurrently [13]. The most common and the most troublesome GI symptom were fecal incontinence and fecal urgency, which often coexisted with sexual and urinary symptoms. Sexual symptoms included fragile vaginal mucous membranes and dyspareunia [4]. Urinary symptoms often included frequent visits to the toilet, leakage, difficulty in feeling the need to empty the bladder, and difficulty to void [12].

During the past decades, the healthcare has mainly focused on optimizing the cancer therapy and to improve survival. But there is still a great unmet need for long-term rehabilitation. Therefore, the oncologic clinic at the University Hospital in Linköping decided to start a highly specialized rehabilitation clinic in 2013 for patients with late adverse events after oncologic treatment. The objective of the clinic was to be a referral center for patients diagnosed with pelvic cancer with severe late adverse events after oncologic treatment. Our center provides a multi-professional caring team with doctors, nurses, physiotherapists, dieticians, and psychologists, all with highly specialized competence to take care of the late adverse events that affects these patients. To our knowledge, this is one of the few institutions that have adopted this multi-professional advanced rehabilitation structure. We were inspired by the Royal Marsden Hospital in London that established a weekly specialized clinic with a gastroenterologist who specifically helped patients with chronic GI symptoms after cancer treatment. The patients referred to this clinic had all been previously treated with pelvic radiotherapy and had severe GI side effects. Data from this clinic showed that patients treated with pelvic radiotherapy experienced a high number of co-existing side effects, where GI side effects were only one of them. Many side effects persisted and had a clear negative effect on the quality of life for these patients [7].

Multiple studies have concluded that improvement in the management of treatment-related late side effects is needed, but few studies have analyzed the toxicity grade of the late adverse that occurs and further have analyzed the type of interventions and the treatment effects of each intervention used to mitigate these symptoms [2, 3, 6, 10, 14].

The aim of this study was to evaluate the toxicity (between visit numbers 1 and 2) of the late adverse events as GI, urologic, and sexual symptoms by using the common terminology criteria for adverse events (CTCAE). Further, we wanted to study different treatment strategies as bile salt sequestrants for diarrhea, anticholinergic drugs for urge to pass urine/frequent visits to the toilet, and local estrogens for vaginal dryness/pain in patients with pelvic cancers who received oncologic treatment.

## Materials and methods

### Patients

This retrospective longitudinal cohort study comprised 90 patients who had at least one visit to the clinic for late adverse events at Linköping university hospital between 2013 and 2019. This center started in 2013 and was devoted to patients who had newly diagnosed side effects more than 3 months after the end of the oncologic treatment or who had remaining symptoms several years after finishing treatment. All women who visited our specialized rehabilitation clinic from 2013 to 2019 were included in this study. The time point between the first visit and the end of the oncologic treatment varied between 7 months up to several decades (At most 29 years, mean 46 months).

In total, 90 patients with pelvic cancer who received some type of oncologic treatment visited the center for late adverse events between 2013 and 2019. The patients had been referred to this clinic from other regions in Sweden, including the county council of Jönköping, Kalmar, Östergötland, Sörmland, and Västmanland. Altogether, they included a population of ~ 1.5 million inhabitants.

The patients in this study received treatment due to the following cancer diagnoses: cervical (*n* = 49), endometrial (*n* = 13), ovarial/tuba (*n* = 6) vulva (*n* = 6), vaginal (*n* = 3), anal (*n* = 7), rectal (*n* = 2), and other cancers (*n* = 4). The treatment included, radiotherapy, both external beam radiotherapy (ERBT) and brachytherapy (BT), surgery and chemotherapy. The treatments were given either in combination or as a single treatment as shown in Table 1. All oncologic treatments were given according to the Swedish national guidelines.

**Table 1:** Characteristics of 90 patients who visited the specialized rehabilitation clinic in Linköping

Variables	*N* (%)
Type of cancer	
Cervical	49 (54.5)
Endometrial	13 (14.4)
Vulva	6 (6.7)
Vaginal	3 (3.3)
Ovarial/tubar/peritoneal	6 (6.7)
Anal	7 (7.8)
Rectal	2 (2.2)
Other	4 (4.4)
Tumor stage	
I	47 (52.2)
II	21 (23.3)
III	13 (14.5)
IV	2 (2.2)
Unknown	7 (7.8)
Type of oncologic treatment	
Radiotherapy	3 (3.3)
Surgery	24 (26.7)
Surgery + chemotherapy	9 (10.0)
Surgery + chemotherapy + radiotherapy	12 (13.3)
Radiotherapy + chemotherapy	33 (36.7)
Surgery + radiotherapy	9 (10.0)
Age at diagnosis (mean) Age* (mean)	48.8 years 53.7 years
Performance status*	
0 1 2	70 (77.8) 12 (13.3) 8 (8.9)
Disease free (cancer)*	
Yes No	88 (97.8) 2 (2.2)
Other diseases**	
Hypertension Diabetes Hypothyroidism Cardiovascular disease Inflammatory bowel disease (IBS)	12 (13.3) 4 (4.4) 4 (4.4) 7 (7.8) 1 (1.1)

*At first visit

**Calculated in percent (%) of the 90 patients

### Data

A review of the patient’s oncologic and surgical records was performed in all patients that had at least one visit at the center for late adverse events between the years 2013 and 2019. The patient’s files were reviewed up to 13 months after their first visit, and the number of visits varied from one to two. Tables 2 and 3 describe the different type of GI, sexual and urologic symptoms, and measures/treatments that were used at our rehabilitation clinic.

**Table 2:** Different type of GI, sexual, and urologic symptoms for the 90 patients who visited our specialized rehabilitation clinic

GI symptoms	Sexual symptoms	Urologic symptoms
Diarreha	Pain at sexual intercourse	Urge to pass urine
Constipation Flatulence	Feeling of dryness/rubbing sensation in vagina	Urinary leakage Hematuria
Melena	Tight and stiff feeling in vagina due to adhesions	Pain during bladder filling/emptying
Mucous in stool Difficulties to empty the bowel	Spasm in the vagina Foul-smelling discharge	Cramping around urethra Difficulty emptying the bladder
Fecal incontinence	Swelling and redness of vulva	Recurrent urinary tract infections
Subileus	Blister and sores in vulva/vagina	
Alternating hard and loose stools	Bleeding	Peeing with scattered stream of urine
	Reduced sex drive/ability to orgasm	
	Feeling of disgust

**Table 3:** Different type of measures/treatments for GI, sexual, and urologic symptoms used for the 90 patients who visited our specialized rehabilitation clinic

Measures/treatment GI symptoms	Measures/treatment Sexual symptom	Measures/treatment Urologic symptoms
Bulking agents (inolaxol, vi-sibin)	Local estrogens	Anticholinergicum
Loperamid	Antibiotics	Local estrogens
Antiemetics Dietician/dietary advice Proton pump inhibitors Pain relivers for pain and cramps (diemtikon, saroten, egazil, papaverine)	Antimycotic treatment Knots and relaxation exercise Vaginal dilatator Discussion on multidisciplinary team meetings (MDK) Counselor	Referral to urologist for cystoscopy Pinch exercises Instillations via urologist with hyaluronic acid preparations Discussion on multidisciplinary team meetings (MDK)
Bile acid sequestrants (cholestagel, lestid, questran)		Hyperbar oxygen treatment
Pancreas enzyme substitution (creon)		
Antibiotics (metronidazole/doxyferm)		
Medicines for rectal bleeding (xyloproct, scheriproct, asacol)		
Referal to gastroenterologist for coloscopy Hyperbar oxygen treatment		

Data of diagnosis, tumor stage, differentiation, performance status, oncologic treatment, and late adverse events were collected from the patients’ medical records. The adverse events were retrospectively graded between 1 and 5 according to the CTCAE grading system [15]. The CTCAE grading scale was chosen as it is the most used system for grading oncologic side effects. Regarding the toxicity grading, grade 0 meant no side effects, grade 1 meant mild side effects, grade 2 moderate side effects, grade 3 severe side effects, grade 4 life-threatening side effects, and grade 5 death related to side effects. No patients in our study had grade 4 or 5 side effects. The grade of symptoms was combined into two subgroups where grade 0–1 was considered as one group and grade 2–3 as the other group. Each intervention used as a treatment for the adverse events was documented. Sexual symptoms were determined and treatment with local estrogens prescribed by anamnestic and clinical findings and by a gynecological exam by an experienced senior specialist in gynecologic oncology. GI and urinary symptoms were determined by anamnestic findings and gynecological exam. Bile salt sequestrants and anticholinergic treatment were prescribed due to anamnestic findings without other investigation.

All the patients who visited the center for late adverse events met the same doctor at both visit numbers 1 and 2. Some patients also met a dietician for dietary advice regarding their GI symptoms and a psychologist regarding their sexual symptoms and general well-being. The patients also had regular follow ups by phone by a nurse working at the rehabilitation center. The grading of toxicity for each patient was performed by two independent researchers’ (S. Iselius and A. Holmqvist). The study protocol was approved by the regional ethical committee in Stockholm, Sweden (Reference number: 2021–05,034) and was in accordance with the Declaration of Helsinki. All patients have signed a written consent form.

### Statistical analyses

The chi-square method and the Fischer’s exact test was used to study the differences in the frequency and the toxicity of side effect between visit numbers 1 and 2. All comparisons were performed by using matched cases. The tests were two-sided and *P* value of *P* < 0.05 was considered statistically significant.

## Results

### Patients

Ninety patients with pelvic cancer who received oncologic treatment participated in the study. Patients with several different types of cancers were included. Most of the patients (85%) were diagnosed with gynecological cancer. The treatment included surgery, radiotherapy, and chemotherapy in different treatment combinations where 63.3% of the 90 patients received radiotherapy (Table 1).

Most of the patients that received oncologic treatment were in stage I (52.2%), 23.3% were in stage II, and 24.4% in stage III or IV. The tumor stage was unknown (due to no register data) for 7.8% of the patients. The mean age of the patients was 48.8 years (range 19–82) and the mean age at the first visit was 53.7 (range 19–86). Few patients had other diseases at the time for diagnosis (Table 1).

### Side effects

We studied the panorama of late adverse events as GI, sexual, and urinary symptoms in the patients referred to our clinic. Our results showed that of all the patients referred to our clinic, 74.4% (*N* = 67) had GI symptoms, 55.5% (*N* = 50) sexual, and 37.8% (*N* = 34) urinary symptoms (Table 4). Two or more adverse events were present concurrently in 81.6% of the patients. Patients receiving radiotherapy experienced a higher frequency of late adverse events compared to patients who had surgery alone or surgery in combination with chemotherapy as shown in Table 4. In the group receiving radiotherapy 84.2% experienced GI side effects, 64.9% had sexual symptoms and 45.6% presented with urinary symptoms. Most patients were free from side effects in the group with only surgery (Table 4).

**Table 4:** Late adverse events as GI, sexual, and urinary symptoms occurring because of different oncological treatments

Visit number 1 (*N* = 90)	Treatment		
Side effects	Surgery	Surgery + chemotherapy	Radiotherapy ± surgery ± chemotherapy
	*N* = 24	*N* = 9	*N* = 57
GI symptoms	Yes, 12 (50.0%)	Yes, 7 (77.8%)	Yes, 48 (84.2%)
	No, 12 (50.0%)	No, 2 (22.2%)	No, 9 (15.8%)
Sexual symptoms	Yes, 9 (37.5%)	Yes, 4 (44.4%)	Yes, 37 (64.9%)
	No, 15 (62.5%)	No, 5 (55.6%)	No, 20 (35.1%)
Urinary symptoms	Yes, 5 (20.8%)	Yes, 3 (33.3%)	Yes, 26 (45.6%)
	No, 19 (79.2%)	No, 6 (66.7%)	No, 31 (54.4%)

### Difference in toxicity of the adverse events between different visits

Further, we analyzed the changes in toxicity of the side effects as GI, sexual, and urinary symptoms by comparing the frequency of symptoms between visit numbers 1 and 2 (Table 5). Here, we showed that 36.6% had a reduction in GI symptoms (*P* = 0.013), 18.3% in sexual symptoms (*P* < 0.0001) and 15.5% in urinary symptoms (*P* = 0.004) at visit 2 compared to visit 1 (Table 5). Only 4.2% had an increase in GI symptoms, 1.4% in sexual symptoms and 5.6% an increase in urologic symptoms between visit numbers 1 and 2 as shown in Table 5.

**Table 5:** Change in toxicity grade of GI, sexual, and urinary symptoms between visit number 1 and 2 in 90 cancer patients who visited the specialized rehabilitation center in Linköping

Change in CTCAE toxicity	Visit 1 vs. 2	*p* value
GI symptoms	*N* = 71 (%)	
Less grade of symptoms (2–3 to 0–1)	26 (36.6)	0.013
Increased grade of symptoms (0–1 to 2–3)	3 (4.2)	
Unchanged grade 2–3 symptoms	14 (19.7)	
Unchanged grade 0–1 symptoms	28 (39.4)	
Sexual symptoms	*N* = 71 (%)	
Less grade of symptoms (2–3 to 0–1)	13 (18.3)	< 0.0001
Increased grade of symptoms (0–1 to 2–3)	1 (1.4)	
Unchanged grade 2–3 symptoms	13 (18.3)	
Unchanged grade 0–1 symptoms	44 (62.0)	
Urinary symptoms	*N* = 71 (%)	
Less grade of symptoms (2–3 to 0–1)	11 (15.5)	0.004
Increased grade of symptoms (0–1 to 2–3)	4 (5.6)	
Unchanged grade 2–3 symptoms	7 (9.9)	
Unchanged grade 0–1 symptoms	49 (69.0)	

*All 71 cases were matched and analyzed by chi-2-test and Fisher exact test. Grade 0 = no side effects, grade 1 = mild side effects, grade 2 = moderate side effects, grade 3 = severe side effects

### Interventions

**Fig. 1:**
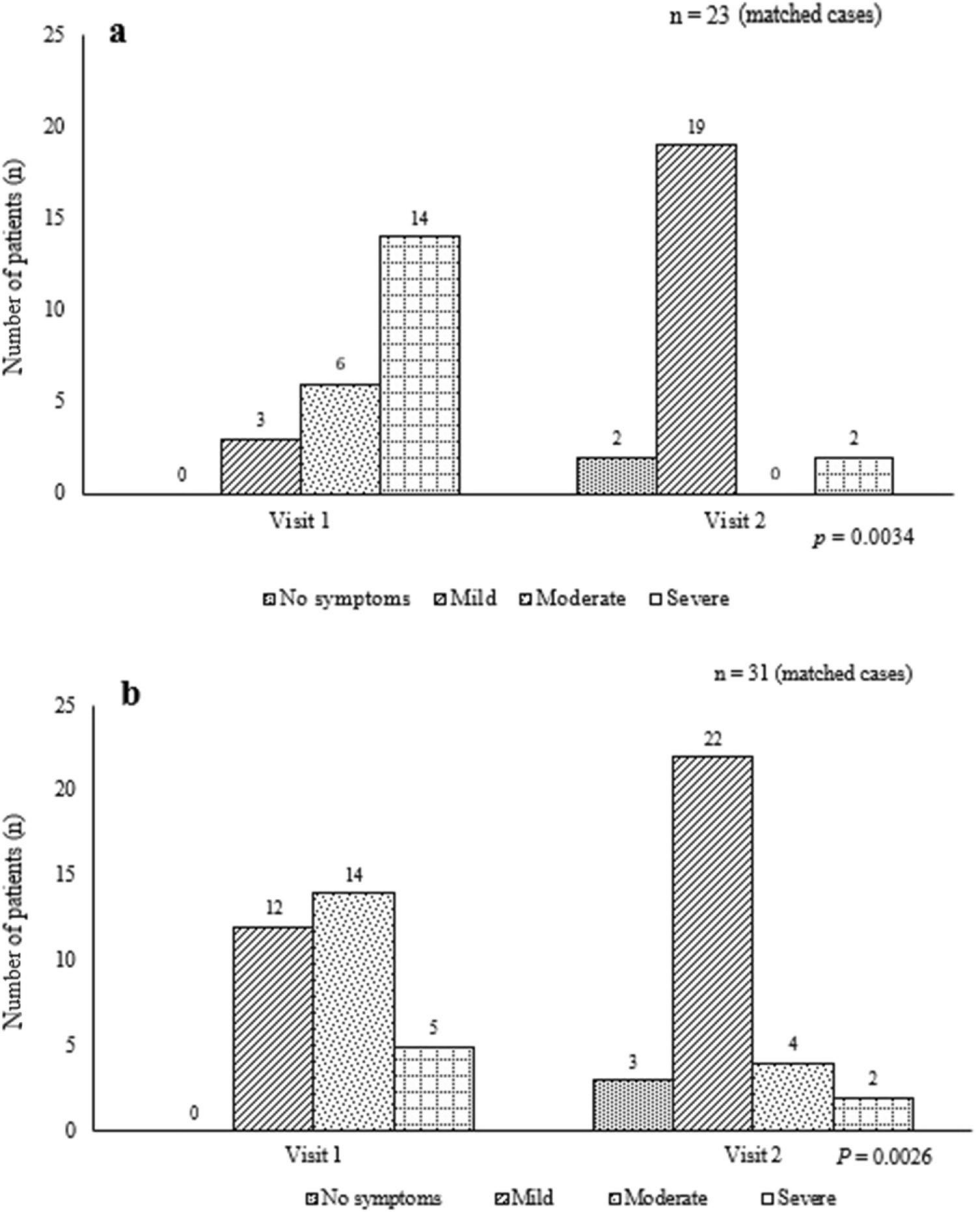
Change in toxicity (grade) of GI side effects as diarreha after administration of bile acid sequestrants between visit numbers 1 and 2 (**a**). Change in toxicity of sexual side effects as vaginal pain/dryness after administration of local estrogens between visit numbers 1 and 2 (**b**)

Patients received clinical consultation and treatment by an experienced oncologist at each visit. In case of more complicated findings, specialists in other fields were consulted and further/additional diagnostic measures were taken to rule out other illnesses or relapses of cancer. Twenty-four patients (30.3%) with severe diarrhea/fecal incontinence or other GI symptoms were prescribed bile salt sequestrants. Almost all patients (23 out of 24 patients) were still on medication at the follow-up visit. Interestingly, out of these 23 matched cases, 21 patients (91.3%) showed a significant improvement in toxicity of GI symptoms at the follow-up visit compared to the first visit (*P* = 0.0034, Fig. 1a).

Fifty patients had sexual symptoms at visit number 1, and out of these 50 patients, 36 patients (72.0%) were prescribed local estrogens. Thirty-one of these 36 patients were matched cases (came to both visits 1 and 2). Here, we showed that the sexual symptoms were improved in 58.1% (*n* = 18) of the 31 patients after applying local estrogens. Thirteen patients (41.9%) did not have any effect of the treatment. None got worse after having local estrogens (*P* = 0.0026, Fig. 1b).

Next, the urinary symptoms as urge to pass urine and frequent visits to the toilet were studied. Only 2 (2.2%) patients out of 90 received anticholinergic treatment at visit 1, and only one of these patients came to visit number 2. No further analysis was possible to perform in the group with anticholinergic treatment as there were too few patients.

### Time elapsed before occurrence of side effects

**Fig. 2:**
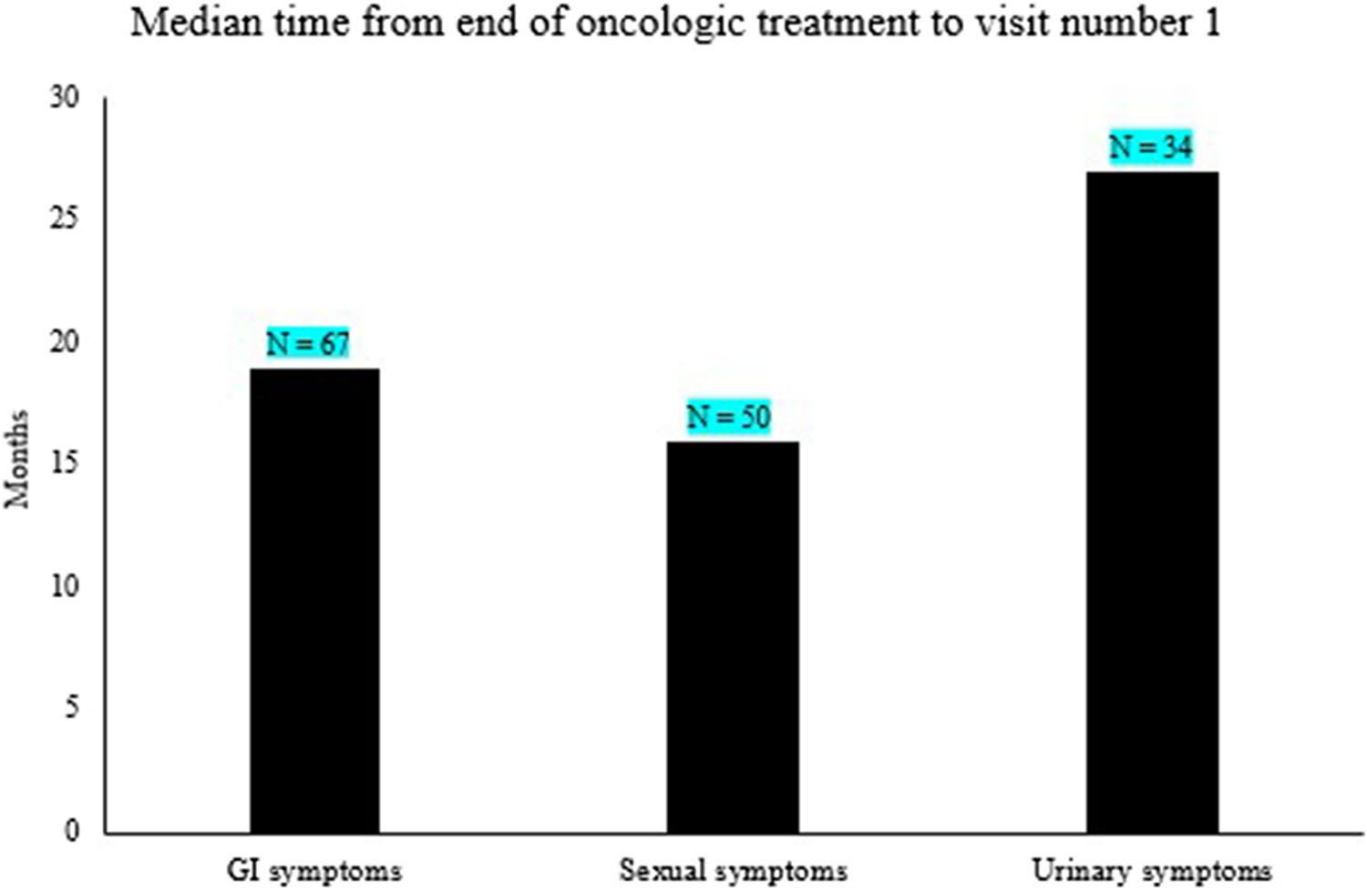
GI, sexual, and urologic side effects, median time from end of oncologic treatment to the first vistit at the rehabilitation clinic in Linköping

Further, we studied the time elapsed (months) between end of treatment and the first visit at our referral clinic. In general, the patients visited the clinic for the first time within a mean time of 21 months (range 2–345) after the end of treatment. The mean time for occurrence of GI side effects after finishing treatment was 19 months (range 2–340, *N* = 67), for sexual symptoms 16 months (range 2–345, *N* = 50), and for urinary symptoms 27 months (range 2–345, *N* = 34, Fig. 2). It seems like the urinary symptoms appears later than the GI and sexual symptoms as shown in Fig. 2.

## Discussion

Several recent studies have analyzed the relationship between oncologic treatment and survival in pelvic cancer patients. However, few studies have evaluated if the late adverse events as GI, sexual, and urologic symptoms can be managed more optimally at a specialized, multi-disciplinary rehabilitation clinic. Further, few studies have evaluated the effect of various pharmacological interventions in relation to the adverse events that develop after oncologic treatment in pelvic cancer patients [4, 6]. All patients (only women) included in this study visited our highly specialized oncologic rehabilitation clinic at the University hospital in Linköping from 2013 to 2019.

A comparison of the frequency of side effects between the first visit and the follow-up visit at our specialized oncologic rehabilitation clinic showed a significant reduction in GI, sexual, and urologic symptoms (36.6%, 18.3%, and 15.5%, respectively). Only 4.2% had increased GI symptoms, 1.4% had sexual symptoms, and 5.6% had increased urologic symptoms between visit numbers 1 and 2. As far as we know, this is the first study which has evaluated the change in toxicity/frequency of symptoms of late side effects between two visits after oncologic treatment in pelvic cancer patients. Further, our study demonstrated that the late adverse events as GI, sexual, and urinary symptoms were significantly reduced at the follow-up visit at our specialized rehabilitation clinic.

We also showed that out of the pelvic cancer patients referred to our clinic, 74.4% experienced GI side effects, 55.5% had sexual side effects, and 37.8% had urinary symptoms such as urinary urgency and leakage after oncologic treatment. There is no previously published report, which has studied a similar patient cohort with cervix cancer and evaluated the frequency of side effects as GI, sexual and urinary symptoms. In one study by Gillespie et al. (2007), the frequency of GI side effects in pelvic cancer patients who visited a specialized rehabilitation clinic in London (UK) was studied by using questionnaires before referral to a consultant gastroenterologist. This particular study showed that around 70–80% of the women and 40–80% of the men experienced GI symptoms as diarrhea and fecal urgency [7]. Thus, we can conclude that the frequency of GI, sexual, and urinary symptoms are high in this selected group of cancer patients who have received curative pelvic radiotherapy.

Our specialized rehabilitation clinic takes care of patients that have been referred from other clinics located in our catchment area who do not have the ability to handle patients with the complex symptomatology of severe late adverse events. Our center provides a multi-professional care team with doctors, nurses, physiotherapists, dieticians, and psychologists, all with highly specialized competence to take care of these late side effects. The treatment for each patient is individual and dependent on the type of side effects present in individual patients. All patients meet an oncologist either alone or together with other healthcare professionals such as nurses, physiotherapists, dieticians, curators. Individual cases are often discussed at a multidisciplinary team conference where other specialties including gynecology, urology, gastroenterology, and anesthesia (working with pain) participate. The doctor working at our clinic usually initiates the necessary treatment for the patient and depending on the type/grade of side effects they can be further referred to other specialties with additional competence to help the patient. Most patients referred to this clinic have a complex symptomatology (81% of the patients have 2 or more side effects at the same time), often with a combination of late adverse events such as GI, sexual, urologic, lymphedema, and psychological problems. They are, thus, a more selected group of patients, which could partly explain the slightly higher frequency of GI and sexual symptoms observed in our study compared to others. Also, differences in the type of treatment modalities could explain the differences in frequency of symptoms. In our patient cohort, there were fewer patients who received curative RT and more patients underwent surgery alone compared to other studies [4, 5, 9, 12]. Also, the median time from finishing treatment to the study measurement could be the reason for differences in frequency of symptoms.

In line with previous reports, our patients experienced multiple late adverse events at the same time, where 81.6% of the patients had two or more adverse events simultaneously which reflects the complexity of symptomatology for these patients [13, 16].

There was a large variation between different patients in the time point for being referred to our clinic after oncologic treatment. Some patients had their first visit just 2 months after having finished their treatment and others many years later. Different adverse events seem to start at different time points after oncologic treatment. The GI and sexual symptoms seem to occur earlier after diagnosis with a median time of 19 and 16 months whereas the urinary symptoms were more common later with a median of 27 months after diagnosis. The later appearance of urinary symptoms has also been described in previous reports [5, 11]. Thus, our results suggest that GI and sexual symptoms appear earlier compared to the urinary symptoms after oncologic treatment in pelvic cancer patients.

Further, we specifically evaluated the effect of treatment with bile salt sequestrants in patients with severe GI side effects including diarrhea and fecal incontinence. Ninety-one percent of the patients who were prescribed bile salt sequestrants had an improvement in grade of symptoms at their follow-up visit compared to their first visit. As far as we know, only one previous study has analyzed the treatment effect of bile salt sequestrants in cancer patients. In this study, 87 patients (33%) with various types of cancers were diagnosed with bile salt malabsorption (BAM) using a Selenium Homocholic Acid Taurine (SeHCAT) scan. In line with our results, 85% of the patients in this study diagnosed as having BAM had a beneficial effect of treatment with bile salt sequestrants [6], suggesting that bile acid sequestrants significantly improves the late side effect as diarrhea/fecal incontinence in pelvic cancer patients with oncologic treatment.

Further, we continued to investigate the potentially beneficial effect of treatment with local estrogens in patients with sexual symptoms as vaginal dryness and pain. Here, we showed that 58% of the patients had an improvement of their sexual symptoms after local estrogen application. Forty-one patients did not have any effect at all, and no patients had an increase in symptoms with treatment. As far as we know, no previous study have compared the treatment effect of local estrogens in cancer patients between two clinical visits. One study showed that estrogen receptors (ERs) were reduced in the vaginal mucosa in cancer survivors after pelvic RT compared to healthy controls [8]. Others showed that local estrogens were used more frequently in the women with cervical cancer compared to the control group [4]. In a study on healthy postmenopausal women, it was shown that 60% had a treatment effect of local estrogens [3] which is the same level of treatment effect as in our study where 58% was shown to have reduced side effects with local estrogens. Thus, our results suggest that the majority of the pelvic cancer patients with oncologic treatment have a beneficial effect of treatment with local estrogens. Therefore, local estrogens should be recommended as treatment for sexual symptoms in most pelvic cancer patients with oncologic treatment.

This highly specialized clinic for late adverse events is unique in Sweden, and our study suggests that establishing similar clinics at other hospitals could make a difference for these patients. Today, most of the cancer patients survive their disease and there will therefore be an increasing number of patients who suffer from late side effects after cancer treatment. Gillespie et al. (2007) concluded that a specialist evaluation and management for chronic side effects is really needed [7]. It is also clear that pharmacological intervention with bile salt sequestrants and local estrogens has an ameliorative effect and provides symptomatic relief.

A potential weakness in this study could be that the grading of symptoms was performed retrospectively by the author, and no formula of patient-reported outcome measurements was used to grade the patients’ own experience of side effects. This study was retrospective and based on a relatively small cohort of patients and the evaluation of the oncologic treatment was carried out using the patients’ medical records. Although our study is small, we consider it to be important, as few studies have focused on evaluating the effects of treatment interventions to mitigate late adverse events. Our study shows that a significant improvement can be made in the clinical care of patients experiencing late adverse events by reducing the toxicity of side effects.

In conclusion, the late adverse events as GI, sexual and urinary symptoms were significantly reduced between the first and the follow-up visit at the specialized rehabilitation clinic in Linköping. Bile salt sequestrants and local estrogens were shown to be effective treatments for side effects as diarrhea and vaginal dryness/pain. In the future, similar specialized rehabilitation centers need to be established and may play an important role in reducing the side effects and improving quality of life for long-term cancer survivors after oncologic treatment.

## References

[1] Abayomi J, Kirwan J, Hackett A (2009). The prevalence of chronic radiation enteritis following radiotherapy for cervical or endometrial cancer and its impact on quality of life. Eur J Oncol Nurs.

[2] Andreyev HJ (2007). Gastrointestinal problems after pelvic radiotherapy: the past, the present and the future. Clin Oncol (R Coll Radiol).

[3] Bannaga A, Kelman L, O'Connor M, Pitchford C, Walters JR, Arasaradnam RP (2017). How bad is bile acid diarrhoea: an online survey of patient-reported symptoms and outcomes. BMJ Open Gastroenterol.

[4] Bergmark K, Avall-Lundqvist E, Dickman PW, Henningsohn L, Steineck G (1999). Vaginal changes and sexuality in women with a history of cervical cancer. N Engl J Med.

[5] de Boer SM, Nout RA, Jurgenliemk-Schulz IM, Jobsen JJ, Lutgens LC, van der Steen-Banasik EM, Mens JW, Slot A, StenfertKroese MC, Oerlemans S, Putter H, Verhoeven-Adema KW, Nijman HW, Creutzberg CL (2015). Long-term impact of endometrial cancer diagnosis and treatment on health-related quality of life and cancer survivorship: results from the randomized PORTEC-2 trial. Int J Radiat Oncol Biol Phys.

[6] Gee C, Fleuret C, Wilson A, Levine D, Elhusseiny R, Muls A, Cunningham D, Kohoutova D (2021) Bile acid malabsorption as a consequence of cancer treatment: prevalence and management in the national leading centre. Cancers (Basel) 13:6213. 10.3390/cancers13246213 10.3390/cancers13246213 PMC8699462 34944833

[7] Gillespie C, Goode C, Hackett C, Andreyev HJ (2007). The clinical needs of patients with chronic gastrointestinal symptoms after pelvic radiotherapy. Aliment Pharmacol Ther.

[8] Hofsjo A, Bohm-Starke N, Bergmark K, Masironi B, Sahlin L (2019). Sex steroid hormone receptor expression in the vaginal wall in cervical cancer survivors after radiotherapy. Acta Oncol.

[9] Jensen NBK, Potter R, Kirchheiner K, Fokdal L, Lindegaard JC, Kirisits C, Mazeron R, Mahantshetty U, Jurgenliemk-Schulz IM, Segedin B, Hoskin P, Tanderup K, Group EC (2018). Bowel morbidity following radiochemotherapy and image-guided adaptive brachytherapy for cervical cancer: Physician- and patient reported outcome from the EMBRACE study. Radiother Oncol.

[10] Jensen NBK, Pötter R, Kirchheiner K, Fokdal L, Lindegaard JC, Kirisits C, Mazeron R, Mahantshetty U, Jürgenliemk-Schulz IM, Segedin B, Hoskin P, Tanderup K (2018). Bowel morbidity following radiochemotherapy and image-guided adaptive brachytherapy for cervical cancer: Physician- and patient reported outcome from the EMBRACE study. Radiother Oncol.

[11] Leddy LS (2018). Management of lower urinary tract symptoms after pelvic radiation in females. Curr Urol Rep.

[12] Lind H, Waldenstrom AC, Dunberger G, al-Abany M, Alevronta E, Johansson KA, Olsson C, Nyberg T, Wilderang U, Steineck G, Avall-Lundqvist E (2011). Late symptoms in long-term gynaecological cancer survivors after radiation therapy: a population-based cohort study. Br J Cancer.

[13] Muls AC (2014). Acta Oncologica Lecture. Gastrointestinal consequences of cancer treatment and the wider context: a bad gut feeling. Acta Oncol.

[14] Muls AC, Watson L, Shaw C, Andreyev HJN (2013). Managing gastrointestinal symptoms after cancer treatment: a practical approach for gastroenterologists. Frontline Gastroenterol.

[15] SERVICES USDOHAH (2017) Common Terminology Criteria for Adverse Events (CTCAE) Version 5.0

[16] Vistad I, Cvancarova M, Fossa SD, Kristensen GB (2008). Postradiotherapy morbidity in long-term survivors after locally advanced cervical cancer: how well do physicians' assessments agree with those of their patients?. Int J Radiat Oncol Biol Phys.

